# Prepartum skeletal muscle reserves and branched-chain volatile fatty acid supplementation have minimal effects in response to intravenous glucose tolerance tests in periparturient dairy cattle

**DOI:** 10.3168/jdsc.2024-0603

**Published:** 2024-07-14

**Authors:** K.M. Gouveia, L.M. Beckett, M.N. Flinders, T.M. Casey, J.P. Boerman

**Affiliations:** Department of Animal Sciences, Purdue University, West Lafayette, IN 47907

## Abstract

Periparturient dairy cows experience metabolic adaptations to prepare for increased nutrient requirements of the fetus and the onset of lactation. Adaptations include increased peripheral tissue insulin resistance, which can be evaluated experimentally using intravenous glucose tolerance tests (IVGTT). The objective of this study was to determine if prepartum skeletal muscle reserves and supplementation of branched-chain volatile fatty acids (BCVFA) in the prepartum period affected blood glucose, β-hydroxybutyrate (BHB), and insulin concentrations 2 wk prepartum and 1 wk postpartum utilizing an IVGTT. At 42 d before expected calving (BEC), the longissimus dorsi muscle depth was measured from an ultrasound image, and based on muscle depth, cows were assigned to either the high muscle (HM; >4.6 cm, n = 17) or low muscle (LM; ≤4.6 cm, n = 17) group. Cows were randomly assigned to either the branched-chain volatile fatty acid (BCVFA) treatment (fed as 39.1 g/d isobutyrate product; 19.4 g/d isovalerate product; 19.6 g/d 2-methylbutyrate product, all on a DM basis) or control (73.0 g/d soyhull pellets on a DM basis) treatment, which were top-dressed daily. Assignment to muscle group and treatment resulted in a 2 × 2 factorial design and the following 4 combinations: HM-CON (n = 7), HM-BCVFA (n = 10), LM-CON (n = 9), and LM-BCVFA (n = 8). On 14 d BEC and 7 DIM an IVGTT was performed following a 1 h fasting period. Baseline blood samples were taken −15 and −5 min before dextrose administration (250 mg/kg BW); blood was then collected at 12 time points over a 3-h time period. Skeletal muscle reserves had no impact on glucose or insulin response across the IVGTT period, whereas BCVFA supplementation increased glucose area under the curve (AUC) in the prepartum period but had no effect in the postpartum period. Prepartum glucose and insulin AUC were higher than the postpartum glucose and insulin AUC. Findings indicate that muscle reserves in the prepartum period do not affect insulin and glucose clearance in periparturient dairy cows, reflecting no differences in insulin sensitivity in response to IVGTT. Changes observed in glucose and insulin AUC between pre- and postpartum IVGTT reflect normal metabolic adaptations to increased energetic requirements of dairy cows between late gestation and early lactation.

## Highlights

• Glucose tolerance tests can evaluate metabolic adaptations to diets and muscle phenotypes.
• Prepartum skeletal muscle reserves had no impact on glucose or insulin responses.
• Branched-chain volatile fatty acid supplementation had minimal effects on glucose or insulin responses.

## Graphical Abstract

**Figure:**
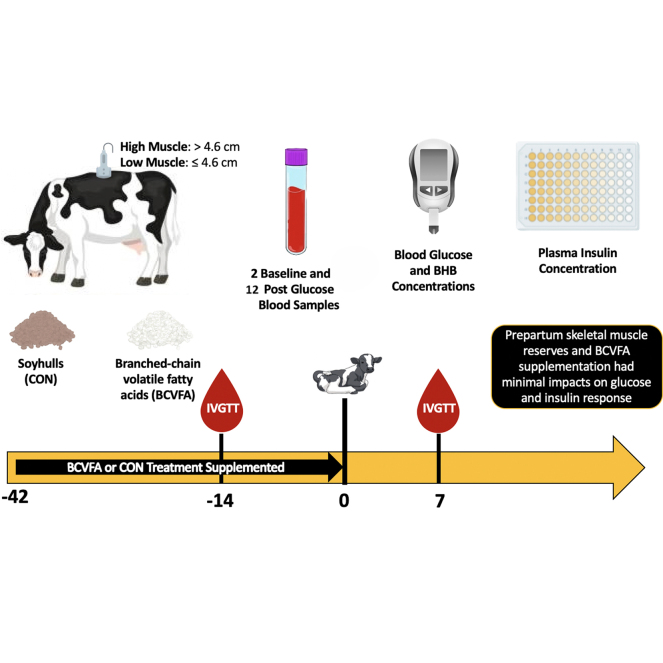
**Summary:** Metabolic adaptations occur in dairy cattle around parturition, partly to spare glucose to support fetal growth during late gestation and milk production in early lactation. The amount of skeletal muscle reserves and branched-chain volatile fatty acid supplementation have the potential to affect glucose supply and, thus, modulate glucose metabolism. However, we found minimal effects of skeletal muscle reserves and branchedchain volatile fatty acid supplementation on insulin and glucose response to intravenous glucose tolerance tests performed at 14 days before expected calving and 7 days in milk. Lactation effects were observed by decreased glucose and insulin concentrations and areas under the curve postpartum, compared with prepartum.

Nutrient and energetic demands of dairy cows are elevated in the periparturient period, due to increased requirements to support fetal growth in late gestation and milk production in early lactation (Bauman and Currie, 1980). Coordinated adaptation of metabolic and physiological processes occur during this time to spare glucose for fetal growth and milk production, through increased glucose production and decreased utilization of glucose in peripheral tissues due to an increase in insulin resistance (Bauman, 2000; Baumgard et al., 2017). Negative energy and protein balance in periparturient dairy cows stimulates the mobilization of adipose and skeletal muscle tissues to meet increased requirements, which is mediated in part by the increased insulin resistance (Komaragiri and Erdman, 1997; Bünemann et al., 2019), with skeletal muscle accounting for the majority of insulin-dependent glucose utilization in ruminants (Mann et al., 2016). Although increased lipolysis closes energy gaps, high rates of fat mobilization are linked to the development of metabolic diseases, such as ketosis, as well as changes in insulin response (Weber et al., 2016; De Koster et al., 2017). Less is known about the impact of muscle mobilization; however, it likely plays a role in insulin and glucose response as it results in the release of AA, which can then be used as substrates for gluconeogenesis, protein synthesis, and ketogenesis (Aschenbach et al., 2010). Our previous study found prepartum skeletal muscle reserves affected insulin sensitivity both pre- and postpartum (McCabe, 2021). Cows categorized as having high skeletal muscle reserves prepartum exhibited reduced insulin sensitivity compared with low muscle (**LM**) reserve cows, as measured with an intravenous glucose tolerance test (**IVGTT**), indicating that the amount of prepartum skeletal muscle may affect insulin response in periparturient cows. Reduced insulin sensitivity in the transition period has the potential to allocate more glucose toward the placenta and mammary gland because of reduced insulin-dependent glucose uptake by other tissue.

Branched-chain volatile fatty acids (**BCVFA**) are used by bacteria, especially cellulolytic bacteria, in the rumen to form branched-chain AA and branched-chain long-chain fatty acids (Mitchell et al., 2023). In this process, intermediates are formed that can affect the production of tricarboxylic acid (**TCA**) cycle intermediates, VFA, and glucose precursors. In the rumen, BCVFA can be used to produce BCVFA-CoA, which can be converted to products that are used by the TCA cycle to produce intermediates for gluconeogenesis, such as phosphoenolpyruvate (Firkins, 2023). In addition, BCVFA-CoA products can be converted to pyruvate and used to produce propionate, acetate, and butyrate (Firkins, 2023). Several studies have reported impacts to VFA production after BCVFA supplementation, resulting in increases in total VFA production (Liu et al., 2018), decreases in total VFA production (Roman-Garcia et al., 2021), or increases to individual VFA such as propionate (Firkins, 2023). Therefore, BCVFA supplementation could potentially affect glucose metabolism through changes in TCA cycle intermediates and VFA production.

We hypothesized that high muscle (**HM**) cows would have increased insulin resistance, as indicated by elevated insulin concentrations with no change to glucose. In addition, BCVFA supplementation would increase insulin sensitivity, mitigating insulin resistance in HM cows. The objective of our study was to determine the impact of prepartum skeletal muscle reserves and prepartum BCVFA supplementation on insulin and glucose concentrations and clearance rates in response to IVGTT in multiparous dairy cows at 14 d before expected calving (**BEC**) and 7 DIM.

Data for this study were collected as part of a larger experiment (Gouveia et al., 2024) performed at the Purdue University Animal Sciences Research and Education Center dairy unit. All animal procedures described were reviewed and approved by Institutional Animal Care and Use Committee (IACUC) protocol #2109002197 before the start of the study. The trial took place from September 2022 through February 2023, and data were collected from 34 multiparous dairy cows with 2.7 ± 0.9 (mean ± SD) lactations, previous 305-d milk yield of 11,442 ± 1,345 kg, and BW of 750.7 ± 74.0 kg at the time of their enrollment, which was 42 d BEC. One day before enrollment, prepartum muscle depth was determined as described in Gouveia et al. (2024) and based on this depth cows were assigned to either a HM (>4.6 cm) or LM (≤4.6 cm) group. At 42 d BEC cows were randomly assigned to either the BCVFA (39.1 g/d isobutyrate product, 19.4 g/d isovalerate product, and 19.6 g/d 2-methylbutyrate product, fed as calcium salts of BCVFA on a DM basis; Zinpro Corporation, Eden Prairie, MN) treatment or the control (73.0 g/d soyhull pellets on a DM basis) treatment. Treatments were top-dressed once daily on the nonlactating dairy cows' diets in the prepartum period only. Following parturition, cows were fed a common early lactation diet. For information regarding both diets see Gouveia et al. (2024). The 2 × 2 factorial study resulted in the following groups: HM-CON (n = 7), HM-BCVFA (n = 10), LM-CON (n = 9), and LM-BCVFA (n = 8).

At 14 d BEC and 7 DIM, an IVGTT was conducted on each cow. The 14 d BEC and 7 DIM time points were selected to test insulin sensitivity because during this time cattle are expected to exhibit the greatest insulin resistance to accommodate energetic and glucose demands (De Koster and Opsomer, 2013). Animals were weighed the afternoon before the IVGTT to determine the amount of dextrose to administer. With the use of local anesthetic (0.03 mg/kg 2% lidocaine; VetOne, Boise, ID), a catheter was inserted in a jugular vein the morning of the IVGTT procedure (0800 to 1000 h). At 1100 h, feed was removed to begin a 1 h fasting period, before dextrose administration. Throughout the collection period, animals had access to water, but feed was withheld until after the final collection time point. Before dextrose administration, 2 baseline blood samples were taken at −15 and −5 min via the catheter lines using a 10-mL syringe. Animals were dosed with 250 mg/kg BW of glucose administered in the form of 50% dextrose solution (VetOne) at time point 0. The dosage of dextrose used was based on findings from other studies and deemed sufficient to elicit an insulin response (Pires et al., 2007; Salin et al., 2012; Duplessis and Girard, 2021). Immediately following the administration of dextrose, catheter lines were flushed with 10 mL of saline, then 10 mL of blood was drawn and discarded before another 10 mL of blood was drawn as the sample. Following blood collection, the lines were flushed with 6 mL of heparinized saline (2 IU/mL heparin concentration in 0.9% saline). Blood samples were collected from the jugular catheters at 0, 5, 10, 15, 20, 30, 45, 60, 90, 120, 150, and 180 min relative to the dextrose administration.

A portion of the whole blood sample was used to measure glucose and BHB concentrations with the Centrivet Glucose and Ketone Reader (Akon Laboratories, San Diego, CA) immediately after blood was collected. Blood was transferred from the syringe to a 10-mL EDTA tube (Becton, Dickinson and Company, Franklin Lakes, NJ) and centrifuged at 4,000 × *g* for 15 min at 4°C. After centrifugation, aliquots of the plasma were frozen at −20°C until further analysis. Plasma concentrations of insulin were measured in duplicate using bovine ELISA insulin kits (ALPCO, Salem, NH) following the manufacturer's protocol. The interplate CV for the insulin plates was 4.75%, and the intraplate CV was 6.50%.

The area under the curve (**AUC**) was calculated for glucose and insulin concentrations from the time points 0 to 180 min using the trapezoidal rule (Pires et al., 2007). Insulin clearance rate was calculated using the following equation:

$$insulinclearancerate=\frac{\ln\;\left({\left[insulin\right]}_{0}\right)-\ln\;\left({\left[insulin\right]}_{60}\right)}{60}.$$

Glucose clearance rate was calculated using the same equation as insulin clearance rate, but with the glucose concentrations used as detailed in Kerestes et al. (2009). Glucose time to half maximal concentration was calculated using the following equation:

$$glucosetimetohalfmaximal=\frac{\ln\;\left(2\right)}{\ln\;\left(glucoseclearancerate\right)}.$$

The quantitative insulin sensitivity check index (**QUICKI**) was calculated using the following equation according to Katz et al. (2000).

$$QUICKI=\frac{1}{\log\;\left(glucose\right)+\log\;\left(insulin\right)}.$$

Cow sample size was determined based on a previous study evaluating IVGTT in a periparturient dairy cow study, with n = 8 cows per group being sufficient to elicit significant differences in insulin response (McCabe et al., 2021). Statistical analysis for glucose, insulin, and BHB concentrations were performed using the mixed procedure of SAS v.9.4 (SAS Institute Inc.) using the model below with pre- and postpartum samples run separately:

$$Y_{ijklm}=\mu +G_{i}+T_{j}+P_{k}+C{\left(GT\right)}_{ijl}+{\left(G\times T\right)}_{ij}+{\left(G\times P\right)}_{ik}+{\left(T\times P\right)}_{jk}+{\left(G\times T\times P\right)}_{ijk}+e_{ijklm},$$

where *Y_ijklm_* represents the dependent variable, and *µ* represents the overall mean, *G_i_* represents the fixed effect of group (*i* = HM or LM); *T_j_* represents the fixed effect of treatment (*j* = CON or BCVFA); *P_k_* represents the fixed effect of time point relative to dextrose administration (*k* = 0 to 180 min); *C*(*GT*)*_ijl_* represents the random effect of cow (*l* = 1 to 34) nested within group and treatment; all 2- and 3-way interactions between group, treatment, and time point; and *e_ijklm_* represents the residual error term. Samples taken after dextrose administration were run as repeated measures relative to time, with first-order autoregressive covariance structure used as it resulted in the lowest Akaike information criterion and the Bayesian information criterion values. Baseline samples before dextrose administration were run with a similar model but without the time point variables and interactions associated with time.

Calculations related to glucose and insulin response measures were analyzed using the following model:

$$Y_{ijk}=\mu +G_{i}+T_{j}+{\left(G\times T\right)}_{ij}+e_{ijklm}.$$

Normality of the variables was checked using the univariate procedure of SAS and the Shapiro-Wilk test. Cook's D was used to identify highly influential outliers with a cutoff of 10/n used, which removed less than 4% of individual data points per analyte. Significance and tendency were declared at *P* ≤ 0.05 and 0.05 < *P* ≤ 0.10, respectively. Results are presented as the LSM ± SEM.

Glucose concentrations decreased between the pre- and postpartum IVGTT with glucose AUC at 17,425 (mg/dL × 180 min) versus 14,195 (mg/dL × 180 min) for all cows, respectively (Table 1 and Figure 1A and 1B). Prepartum, there was a tendency for BCVFA to increase glucose AUC (*P* = 0.10) with no other treatment or group effects for glucose-related calculations. Postpartum, there were no group, treatment, or group by treatment effects for glucose-related variables.

**Table 1:** Response to intravenous glucose tolerance tests for glucose, insulin, and BHB metrics pre- (14 d BEC) and postpartum (7 d DIM) for dairy cattle with differing prepartum skeletal muscle reserves (high vs. low muscle) and fed either a CON or BCVFA supplement during the prepartum period^l^

Response variable	High muscle		Low muscle		SEM^3^	*P*-value^2^		
	CON	BCVFA	CON	BCVFA		Group	Treatment	Group × treatment
Prepartum								
Glucose AUC^4^ (mg/dL × 180 min)	17,161	18,232	16,334	17,974	873.8	0.50	0.10	0.72
Glucose clearance rate (%/min)	1.57	1.63	1.57	1.46	0.141	0.52	0.82	0.51
Glucose time to 1/2 maximum (min)	16.65	16.80	16.66	16.67	0.32	0.84	0.79	0.82
Insulin AUC^4^ (μIU/mL × 180 min)	4,230	4,125	4,579	3,572	570	0.85	0.29	0.39
Insulin clearance rate (%/min)	3.31	2.54	2.75	2.81	0.40	0.70	0.34	0.26
QUICKI^5^	0.34	0.35	0.34	0.34	0.01	0.84	0.96	0.49
BHB baseline^6^ (mmol/L)	0.70	0.73	0.74	0.71	0.09	0.90	0.97	0.77
BHB^7^ (mmol/L)	0.65	0.65	0.68	0.65	0.07	0.79	0.77	0.67
Postpartum								
Glucose AUC^4^ (mg/dL × 180 min)	13,621	14,138	14,062	14,960	755	0.37	0.31	0.78
Glucose clearance rate (%/min)	1.92	1.98	1.90	1.71	0.18	0.39	0.70	0.47
Glucose time to 1/2 maximum (min)	17.52	17.64	17.32	17.45	0.42	0.62	0.75	0.99
Insulin AUC^4^ (μIU/mL × 180 min)	1,775	1,884	1,767	1,674	351	0.74	0.98	0.76
Insulin clearance rate (%/min)	4.30	4.17	4.35	3.98	0.45	0.87	0.55	0.78
QUICKI^5^	0.40	0.40	0.39	0.40	0.01	0.22	0.24	0.28
BHB baseline^6^ (mmol/L)	0.70	0.76	0.94	0.83	0.08	0.05	0.76	0.26
BHB^7^ (mmol/L)	0.64	0.69	0.74	0.72	0.08	0.12	0.74	0.35

^1^Cows were assigned to muscle group and treatment 42 d BEC. Depth of muscle for high muscle (HM) cows was >4.6 cm and low muscle (LM) was ≤4.6 cm. BCVFA treatment included isobutyrate, isovalerate, and 2-methylbutyrate in a 2:1:1 ratio, and CON a soyhull pellet. Four combinations of group × treatment resulted, which include HM-BCVFA, HM-CON, LM-BCVFA, and LM-CON and have the following number of cows in each group, respectively (n = 10, n = 7, n = 8, and n = 9).

2 *P*-values associated with group effects (high muscle vs. low muscle) as determined by longissimus dorsi depth at 42 d BEC, treatment effects (control vs. supplementation with BCVFA), and the interaction between group and treatment.

3 Largest standard error of the mean for group × treatment interactions.

4 AUC = area under the curve.

5 QUICKI = quantitative insulin sensitivity check index calculated using the following equation: $\frac{1}{\log\;\left(glucose\right)+\log\;\left(insulin\right)}.$

6 BHB baseline = mean BHB concentrations in the blood samples prior to dextrose administration (−15 and −5 min).

7 BHB = the mean BHB concentrations in the blood samples taken after dextrose administration (0 to 180 min).

**Figure 1:**
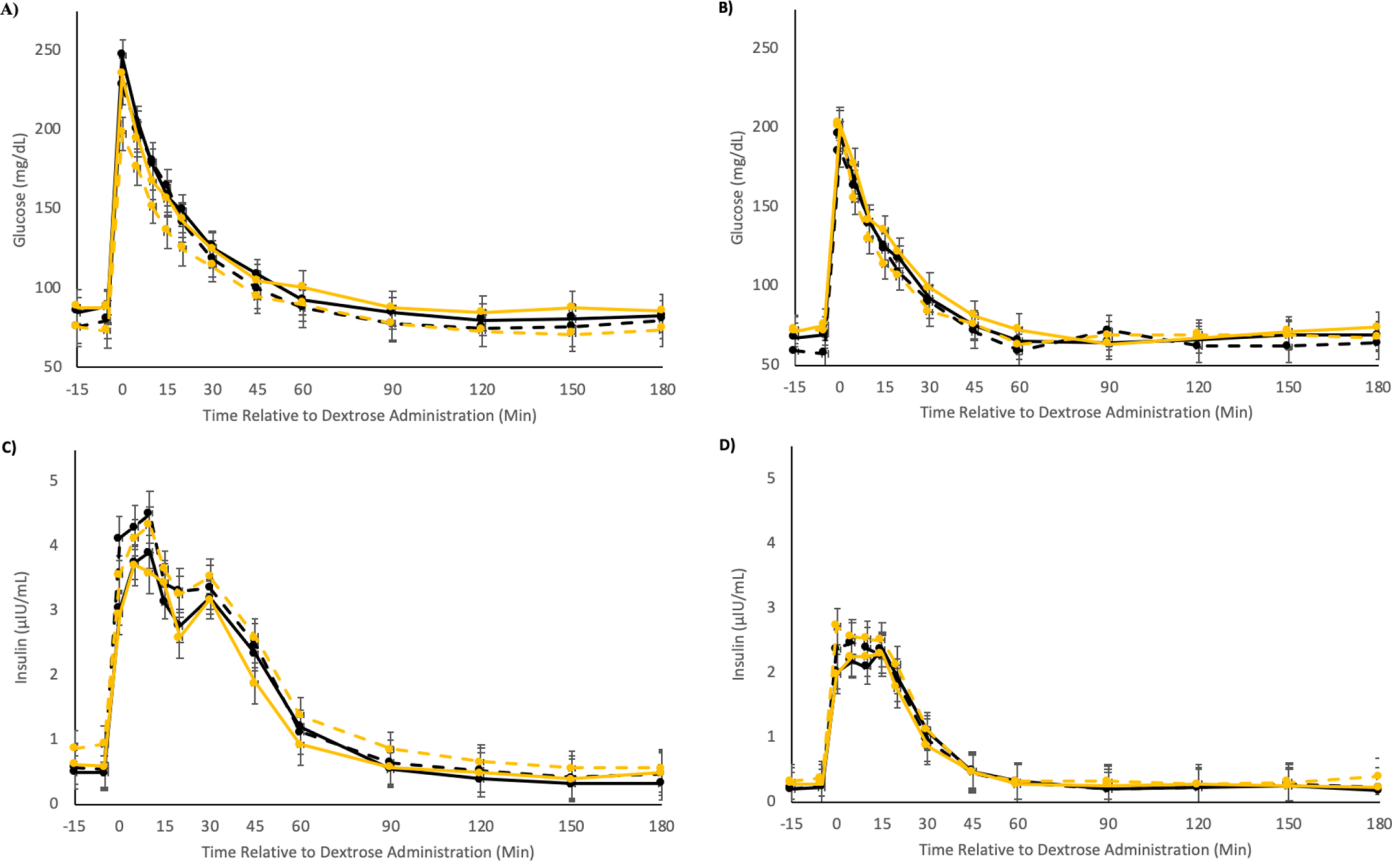
(A) Response curves for blood glucose concentrations 14 d BEC (*P*-values for glucose AUC after dextrose administration are presented in Table 1). Baseline glucose *P*-values (group: *P* = 0.89; treatment: *P* = 0.12; group × treatment: *P* = 0.73). (B) Response curves for blood glucose concentrations 7 DIM (*P*-values for glucose AUC after dextrose administration are presented in Table 1). Baseline glucose *P*-values (group: *P* = 0.23; treatment: *P* = 0.45; group × treatment: *P* = 0.55). (C) Response curves for insulin concentrations 14 d BEC (*P*-values for insulin AUC after dextrose administration are presented in Table 1). Baseline insulin *P*-values (group: *P* = 0.20; treatment: *P* = 0.28; group × treatment: *P* = 0.51). (D) Response curves for insulin concentrations 7 DIM (*P*-values for insulin AUC after dextrose administration are presented in Table 1). Baseline insulin *P*-values (group: *P* = 0.07; treatment: *P* = 0.49; group × treatment: *P* = 0.58) during the IVGTT with HM-BCVFA (solid black line), HM-CON (dashed black line), LM-BCVFA (solid yellow line), and LM-CON (dashed yellow line) dairy cows. Error bars represent the largest SEM for LSM of the group × treatment × time point interaction. HM = high muscle, >4.6 cm; LM = low muscle, ≤4.6 cm; BCVFA = branched-chain volatile fatty acids (isobutyrate, isovalerate, and 2-methylbutyrate); CON = control, soyhull pellets.

Insulin response to IVGTT decreased between pre- and postpartum IVGTT, with insulin AUC 4,126 (µIU/mL × 180 min) prepartum and 1,775 (µIU/mL × 180 min) postpartum (Table 1 and Figure 1C and 1D). Prepartum, there were no group, treatment, or group by treatment effects for insulin-related variables. Postpartum, there were no group, treatment, or group by treatment effects for insulin-related variables except for a group tendency with HM cows having lower insulin concentrations before dextrose administration (*P* = 0.07; Figure 1D).

Prepartum, there were no group, treatment, or group by treatment effects for BHB variables. Postpartum, there were no group, treatment, or group by treatment effects for BHB variables except the baseline BHB concentrations were decreased by 0.20 mmol/L in HM cows versus LM cows before dextrose administration (*P* = 0.05; Table 1).

Based on previous work evaluating insulin response in different muscle phenotype cows (McCabe, 2021), we expected to observe increased insulin resistance in HM cows. Our previous study found that cows with higher muscle reserves had a greater insulin response to IVGTT and no change in glucose concentration, indicating more insulin was required to maintain glucose concentrations. Lack of a difference in IVGTT response between HM and LM group in the current study would indicate that in contrast to our previous findings, prepartum skeletal muscle reserves did not differentially affect factors which mediate glucose homeostasis. This in part could be due to differences in the classification of muscle depth groups between studies, as HM (5.3 vs. 5.0 cm) and LM (4.1 vs. 3.4 cm) averages varied between the current and previous studies, respectively.

Studies investigating BCVFA supplementation have been found to affect VFA production and glucose metabolism (Liu et al., 2018; Roman-Garcia et al., 2021; Firkins, 2023). In the prepartum period, BCVFA supplemented cows tended to have a greater glucose AUC, which could reflect a greater glucose availability. This trend was lost in the postpartum period, potentially due to the greater glucose demands of lactation and the cessation of BCVFA supplementation. Differences in the baseline samples observed in the postpartum period, such as lower insulin and BHB concentrations postpartum in HM cows, may indicate different glucose metabolic adaptations not captured with IVGTT that are affected by skeletal muscle reserves. Despite the lack of an effect of late gestation skeletal muscle reserves and BCVFA supplementation on IVGTT responses, there were dynamic changes in glucose and insulin response curves between pre- and postpartum tests. This clearly demonstrated the metabolic adaptations of periparturient dairy cows as seen in the decreased glucose and insulin AUC from pre- to postpartum, and the increased insulin clearance rate postpartum.

Supplementation of BCVFA has been found to increase the molar proportion of propionate (Firkins, 2023), and while some results are contradictory, there are potential mechanisms for how these VFA changes could alter glucose and insulin response. In Gouveia et al. (2024), weekly glucose differences were observed. The BCVFA cows had higher glucose concentrations both pre- and postpartum compared with CON cows and HM cows had higher prepartum glucose concentrations compared with LM cows. Similar results were not evident in IVGTT responses, and thus do not provide any additional support for a potential mechanism of action. The BCVFA were only provided prepartum, and any postpartum treatment effects would be a result of a carryover effect. It is unclear if the cessation of supplementation has a rapid impact on the rumen environment and VFA production, and if it affected glucose and insulin response.

Few studies have assessed the impact of skeletal muscle on insulin response in dairy cattle; one analyzed insulin signaling proteomes in muscle during an IVGTT in cows fed excess energy prepartum (Mann et al., 2016), whereas another compared high and low muscle phenotype cows' IVGTT responses (McCabe, 2021). Mann et al. (2016) found that skeletal muscle has signaling changes due to parturition and lactation that were not driven by prepartum nutrition differences or lipid metabolism. McCabe (2021) found reduced insulin sensitivity in dairy cattle with greater muscle reserves. Because of the role muscle plays in glucose and insulin in the body, differences between the 2 groups of high and low muscle phenotype cows in our study were anticipated. Although there were minimal differences observed in IVGTT responses, ongoing work in our laboratory indicates there are signaling pathway differences between different muscle phenotype cattle and those supplemented with BCVFA. These differences were not observed during our IVGTT, indicating that IVGTT may not be the most appropriate method to detect these differences in glucose metabolism.

Multiparous dairy cattle with differing skeletal muscle reserves and treatment of BCVFA during the last 6 wk of gestation had minimal differential responses to IVGTT in the pre- or postpartum periods. Prepartum skeletal muscle reserves were found to have no impact on insulin or glucose response following the dextrose dose. Although there was a tendency for increased glucose AUC in BCVFA treatment cows prepartum, during supplementation, this greater AUC was lost postpartum when treatment was not supplemented. These minimal effects indicate that glucose metabolism, as measured through IVGTT, is not largely affected by muscle reserves or supplementation of BCVFA, although both have resulted in improved cattle performance.
